# High current table-top setup for femtosecond gas electron diffraction

**DOI:** 10.1063/1.4983225

**Published:** 2017-05-08

**Authors:** Omid Zandi, Kyle J. Wilkin, Yanwei Xiong, Martin Centurion

**Affiliations:** Department of Physics and Astronomy, University of Nebraska-Lincoln, Lincoln, Nebraska 68588, USA

## Abstract

We have constructed an experimental setup for gas phase electron diffraction with femtosecond resolution and a high average beam current. While gas electron diffraction has been successful at determining molecular structures, it has been a challenge to reach femtosecond resolution while maintaining sufficient beam current to retrieve structures with high spatial resolution. The main challenges are the Coulomb force that leads to broadening of the electron pulses and the temporal blurring that results from the velocity mismatch between the laser and electron pulses as they traverse the sample. We present here a device that uses pulse compression to overcome the Coulomb broadening and deliver femtosecond electron pulses on a gas target. The velocity mismatch can be compensated using laser pulses with a tilted intensity front to excite the sample. The temporal resolution of the setup was determined with a streak camera to be better than 400 fs for pulses with up to half a million electrons and a kinetic energy of 90 keV. The high charge per pulse, combined with a repetition rate of 5 kHz, results in an average beam current that is between one and two orders of magnitude higher than previously demonstrated.

## I. INTRODUCTION

Gas electron diffraction has a long history in the determination of the structure of isolated molecules in the gas phase,^1–3^ in part due to the high scattering cross section of electrons in comparison to x-rays.^4^ There has also been significant progress in time-resolved electron diffraction, which has been used to capture short-lived molecular structures.^5–10^ The field of ultrafast gas electron diffraction (UGED) came to life with the pioneering experiments by the groups of Zewail and Weber in the 1990s and early 2000s that achieved a temporal resolution of a few picoseconds.^11–14^ From then on, the group of Zewail produced a series of ground-breaking electron diffraction results to capture the structures of transient molecular states with very high precision.^15–23^ This was made possible by numerous technological improvements that resulted in a temporal resolution of a few picoseconds combined with high spatial resolution.

A major challenge in UGED is to reach femtosecond resolution while maintaining the high electron beam current needed to capture diffraction patterns with a high signal to noise ratio (SNR). The differential scattering cross section drops rapidly with increasing scattering angle; thus, a high beam current is needed to capture the diffraction pattern at the larger angles that are needed to reach high spatial resolution. Working with highly charged electron bunches requires compensating for the Coulomb effect that leads to broadening of the pulse duration. The development of compact electron guns resulted in a resolution of around 200 fs in ultrafast transmission electron diffraction from solid samples.^24–27^ For UGED, the guns could not be made as compact as those used in condensed matter due to the proximity of the gas source to the accelerator; however, reducing the gun length and the charge in each pulse resulted in a resolution between 0.85 ps and 1 ps.^28–30^ The resolution was limited by both the electron pulse duration and the group velocity mismatch between the laser and electron pulses.^31^ Recent experiments using relativistic electron pulses with MeV energy have reached a resolution of 220 fs.^32–35^ In this case, relativistic effects significantly reduce the Coulomb broadening of the pulse duration and mostly eliminate the velocity mismatch. Operating at these high energies, however, requires a significantly larger infrastructure compared to the table-top electron guns operating at keV energies.

We present here a table-top setup operating at an energy of 90 keV that can reach femtosecond resolution with a high electron beam current. We use a radio frequency (RF) cavity with a longitudinal electric field to compress highly charged electron pulses.^36,37^ The RF compression technology has been used for ultrafast electron diffraction (UED) experiments with solid samples^38–41^ but so far has not been implemented in UGED. We have measured sub-400 fs pulse durations at the position of the sample using a streak camera. The measurement reflects both the electron pulse duration and the time of arrival jitter between the laser and electrons. In addition, our setup has the capability to deliver a tilted laser pulse on the sample to compensate for the group velocity mismatch.^42^ Tilted laser pulses have previously been used by the Zewail group to improve the temporal resolution in ultrafast electron crystallography.^43^ We also show that with the high beam current a high-quality diffraction pattern can be recorded in 20 s, compared to tens of minutes or hours in previous UGED experiments.

In the following, we describe the experimental setup, present measurements of the electron pulse duration, and demonstrate the capability to capture diffraction patterns with a short integration time.

## II. EXPERIMENTAL SETUP

### A. Overview of the experimental setup

**FIG. 1.**
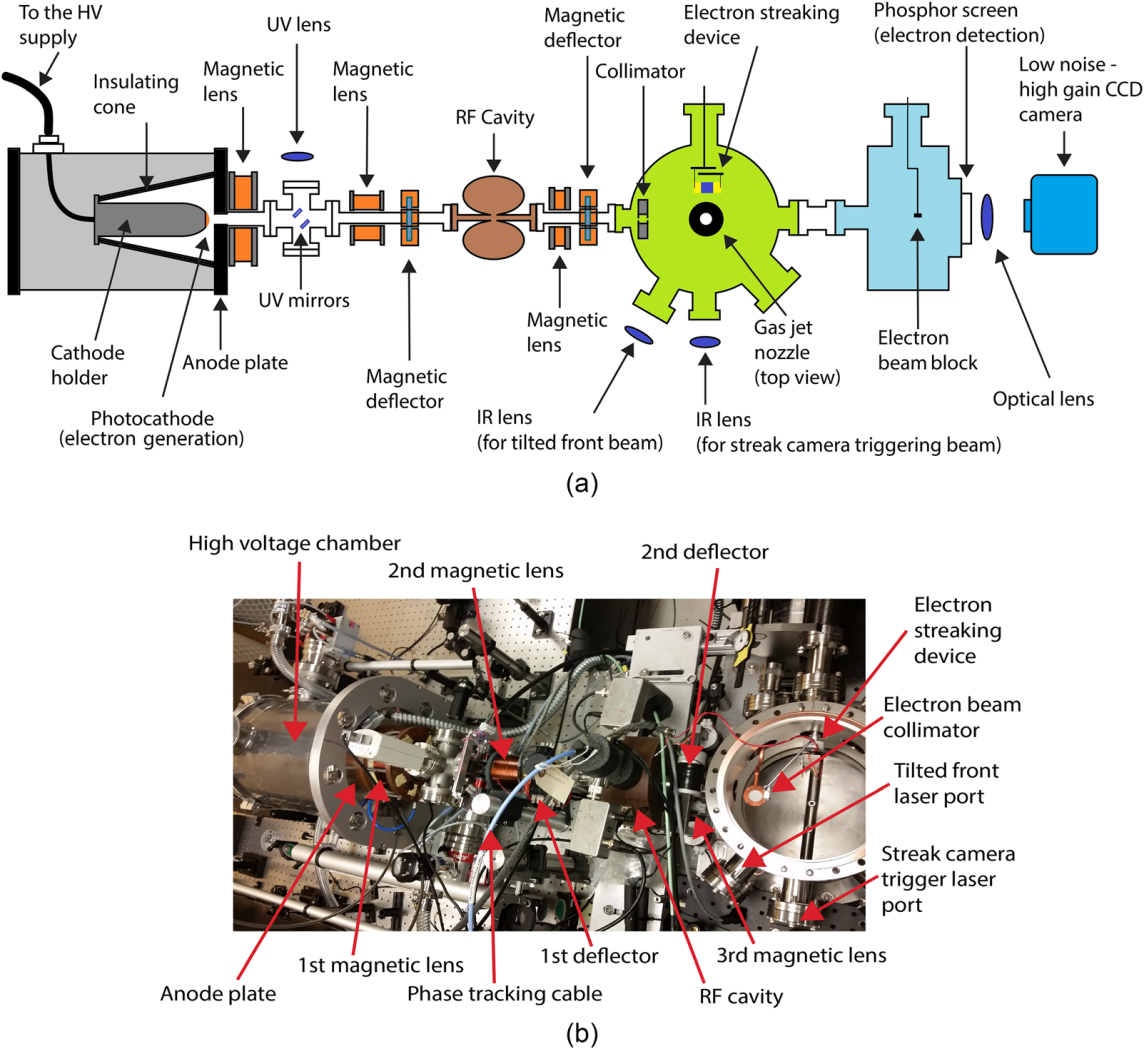
(a) The UGED setup block diagram. The electron pulses are generated by ultrafast laser photoemission and then accelerated in a static electric field. Three magnetic coils are used to control the beam size and an RF cavity is used to temporally focus the beam on the sample. Electrons are collimated and directed to a molecular gas jet target. The scattered electrons are detected by a phosphor screen that is imaged onto the chip of an EMCCD camera. The electron pulse duration is measured with a laser activated streak camera. The streaking device can be moved into the position of the target to measure the electron pulse duration. (b) A photograph of a top view of the UGED setup.

Figures 1(a) and 1(b) depict a block diagram of our UED setup and a photograph of the setup, respectively. The laser system is described in Section II B. The electron pulses are generated in a photoemission process by illuminating the photocathode with femtosecond UV laser pulses. The UV beam is focused by a lens outside the vacuum chamber and directed to the photocathode by an in-vacuum mirror. The reflection from the photocathode is monitored to steer the beam. The photo-emitted electrons are accelerated in a static electric field generated by keeping the cathode at −90 kV. The electron beam exits the acceleration stage through a hole in the grounded anode. We use two magnetic deflectors to steer the electrons along the beamline and three magnetic lenses to control the beam divergence. An RF cavity with a longitudinal time-varying electric field is used to temporally compress the electron pulses at the target position. We have used a homemade laser-activated streak camera to measure the electron pulse duration *in situ*.^44,45^

The target molecules are delivered into the vacuum in a gas jet with a diameter of approximately 200 *μ*m using a small nozzle. The nozzle consists of a 20 mm tube with an inner diameter of 100 *μ*m. A collimating aperture is placed before the sample to remove stray electrons and to match the electron beam diameter to that of the gas jet. The setup includes an optical system to excite the target molecules with laser pulses that are velocity matched to the electrons by the pulse-front tilt technique, which is described briefly in Section II B and in more detail in a previous publication.^42^ The central part of the electron beam is captured by a beam block that is connected to a pico-ammeter to measure the beam current. We have observed that the number of electrons grows linearly with UV laser power up to 5 × 10^6^ electrons per pulse, as expected for a one-photon process.^46^ The scattered electrons are detected using a phosphor screen that is imaged onto a low-noise, high-gain EMCCD camera (Andor, iXon Ultra 888).

### B. Optical system

**FIG. 2.**
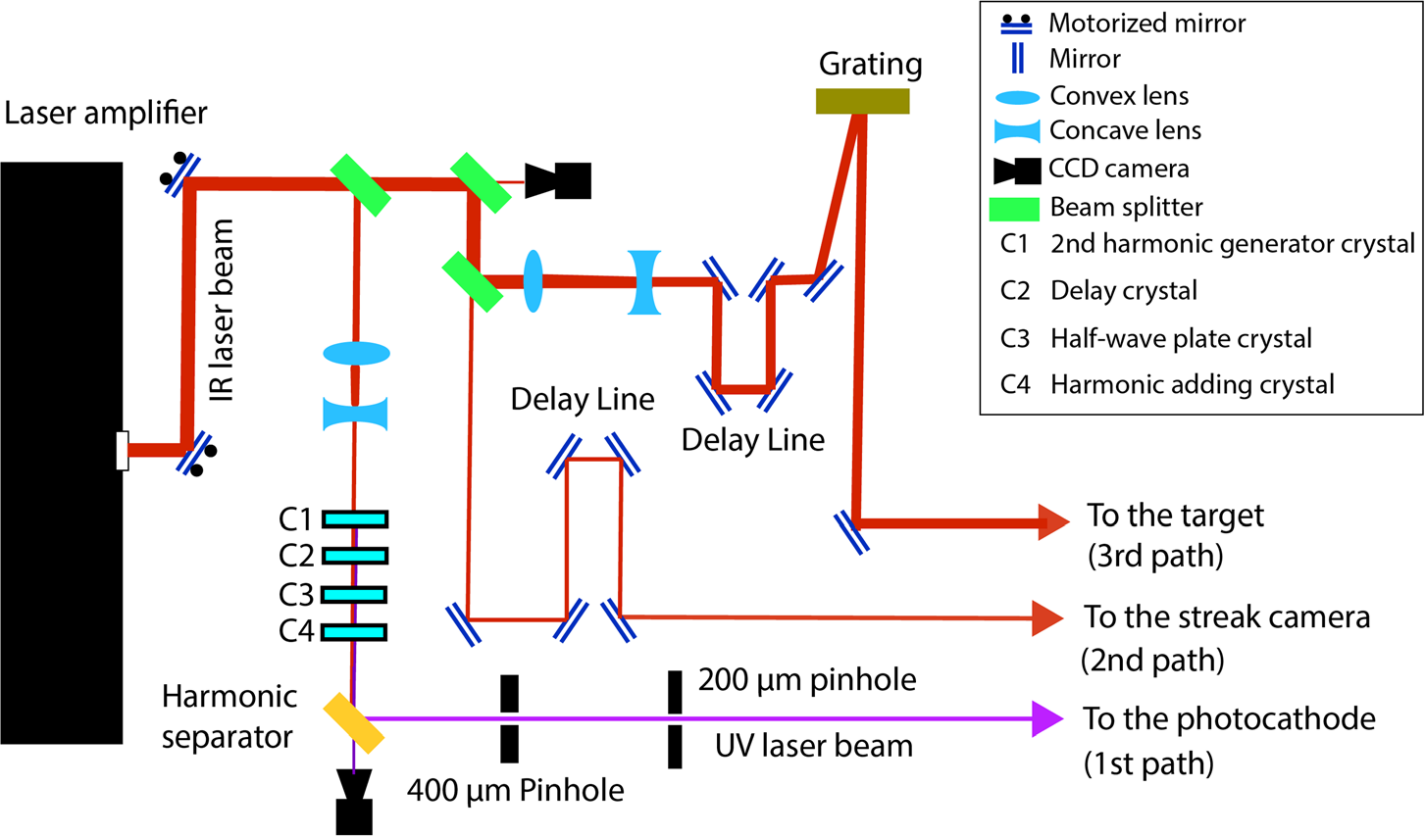
Three laser paths used in the UED setup. The first path consists of nonlinear crystals that convert the IR beam to UV to generate electrons. The second path is used to trigger the streak camera. The third path is designed to tilt the intensity front of the laser beam to velocity match it to the electron beam.

A mode locked Ti:Sapphire laser generates the laser seed pulses with a central wavelength of 800 nm and a repetition rate of 75 MHz. The repetition rate is actively stabilized using a frequency counter that provides feedback to an actuated mirror inside the laser cavity to adjust the cavity length. The laser is amplified using chirped pulse amplification to generate 40 fs, 2 mJ pulses at a 5 kHz repetition rate with a central wavelength of 800 nm and 30 nm bandwidth. The laser beam pointing is actively controlled using a feedback loop with two CCD cameras and two actuated mirrors. The laser beam is split into three paths as shown in Figure 2. The first path is used to trigger the photocathode, the second to trigger the streak camera, and the third is used to excite the sample after going through a pulse tilting setup.

The laser path to the photocathode consists of a frequency tripler that generates 267 nm UV laser pulses. The frequency tripler uses four crystals in an in-line configuration: a BBO second harmonic generator, a delay crystal to temporally overlap the 400 nm and 800 nm pulses, a half-wave plate that matches the polarization of the two pulses, and finally a second BBO crystal that mixes the two pulses to generate the 267 nm laser pulse. After the last crystal, three harmonic separators are used to filter out the IR and blue components. The UV beam is truncated first by a 400 μm diameter pinhole and then by 200 μm one to eliminate remaining beam pointing instabilities. The second pinhole is imaged onto the photocathode by a UV lens with a demagnification of 4 as is shown in Figure 1(a). The laser beam has a uniform circular profile on the photocathode, which results in an emitted electron pulse with a density profile that closely approximates a uniformly charged spheroid,^47^ which can be compressed in all directions by linear forces.

The second laser path is designed to trigger the streak camera that measures the electron pulse duration. Only a small fraction of the laser pulse energy (approximately 80 *μ*J) is needed to trigger the streak camera. The electron pulse duration measurement is described in Section III.

**FIG. 3.**
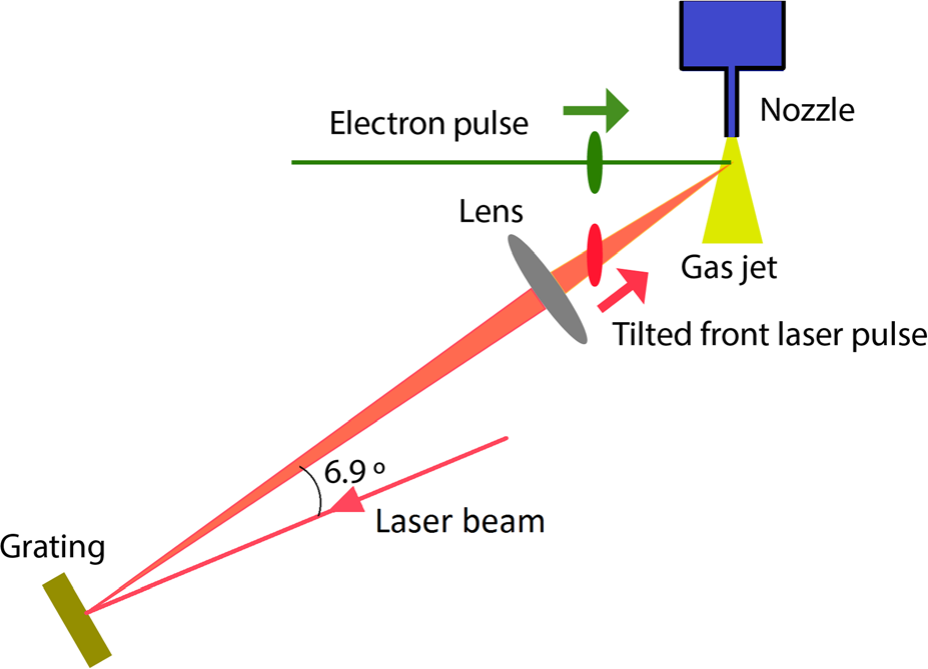
Diagram of the optical setup to deliver laser pulses with a tilted front to compensate the group velocity mismatch. The laser beam is reflected by a grating, which produces a tilted pulse front. The laser beam is incident on the gas target with a 58° angle with respect to the electron beam so that the velocity component in the direction of electron beam propagation matches the speed of the electrons. The grating accordingly tilts the intensity front of the laser beam so that it is parallel to that of the electron beam.

The last path is designed to excite the target and to compensate for the velocity mismatch between the laser and electron pulses. As is shown in Figure 2, an optical delay line is used to match the time of arrival of the laser and electron pulses on the target. A gold-coated holographic grating with grating constant $d=150{\;\text{mm}}^{-1}\;$ tilts the energy front of the laser beam. The tilt angle on the target is determined by the central wavelength of the laser pulse, the angular dispersion of the grating, and the demagnification factor M  of the imaging system that images the grating face onto the target gas jet (Figure 3(a)). The laser beam makes an angle of 58° with respect to the electron beam so that the component of its velocity in the direction of the electron beam matches the speed of the electrons (0.53*c* at 90 keV). The laser is demagnified by a factor 13.5, which results in a pulse front that is tilted to match the electron beam at the sample. The tilted pulse front is compressed temporally only at the image plane of the grating; however, we have previously measured that the pulse duration remains below 100 fs for a length of about 1 mm,^42^ which is significantly longer than our gas target.

The large incidence angle of the pump laser beam with respect to the electron beam means that the laser beam must be larger than the electron beam to excite the whole sample volume that is probed by the electron beam. For example, if both the electron and gas beams have a diameter of 200 *μ*m and the laser beam has a diameter of 300 *μ*m, approximately 90% of the sample will be excited by the laser. The current optical design is optimized for exciting the sample with a laser wavelength of 800 nm. A small change in laser wavelength can be accommodated by changing the angle of incidence of the laser onto the grating and the magnification of the imaging system to deliver the correct tilt angle. For a large change in wavelength, for instance the second or third harmonics of the laser (at 400 nm and 267 nm, respectively), a different grating will be necessary, but the rest of the optical system will not require major changes.

### C. Compression of the electron pulse duration

The electron pulses are compressed by the longitudinal electric field of the RF cavity.^36,37^ When the electrons enter the cavity, there is a decelerating force, and by the time the electrons exit the cavity, the direction of the force has reversed. On the short time scale that it takes the electrons to traverse the cavity, the force changes approximately linearly with time, and hence in the rest frame of the electrons it behaves as a linear force in space. This force produces a temporal focus of the electron pulse at a distance that depends on the amplitude of the electric field. The compression is optimal when the electron pulse is synchronized to the zero-crossing of the electric field. The laser repetition rate of 75 MHz is synchronized to a 3 GHz signal using a synchronizer from AccTec BV, and this signal is amplified using a commercial microwave amplifier and sent to the RF cavity by use of a phase stable coaxial cable. The laser oscillator works as a clock for the whole setup synchronizing the electrons to the RF cavity field. The phase and amplitude of the field in the cavity are adjusted to reach the minimum pulse duration on target.

## III. MEASUREMENT OF THE ELECTRON PULSE DURATION

**FIG. 4.**
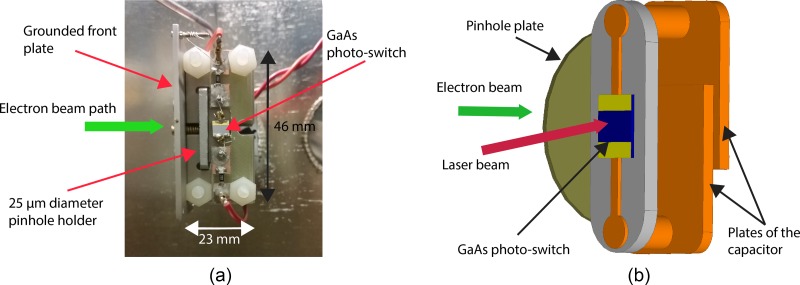
(a) A photograph of the streak camera showing the main components. (b) Diagram of the streak camera. A GaAs photo-switch is placed in parallel to a charged parallel plate capacitor. Once the laser excites the switch, the capacitor is short-circuited resulting in a damped oscillating electric field across the capacitor plates. The electron pulse is first truncated by a pinhole to fit between the capacitor plates. The time-varying electric field introduces a time-dependent deflection to the electron pulse.

We constructed a laser activated streak camera with capability to measure pulse durations below 200 fs (Figure 4). The performance of the streak camera was characterized in a previous publication.^45^ Briefly, the streaking device consists of a charged parallel plate capacitor in parallel to a GaAs photo-switch. Once a laser pulse excites the switch, the capacitor will be short circuited and the electric field across the capacitor starts a damped oscillation. An electron pulse traversing the capacitor will be streaked by the time varying field so that its temporal profile is mapped into the deflection angle. On the detector, the pulse duration can be extracted by recording the beam profiles with the streaking field on and off.

**FIG. 5.**
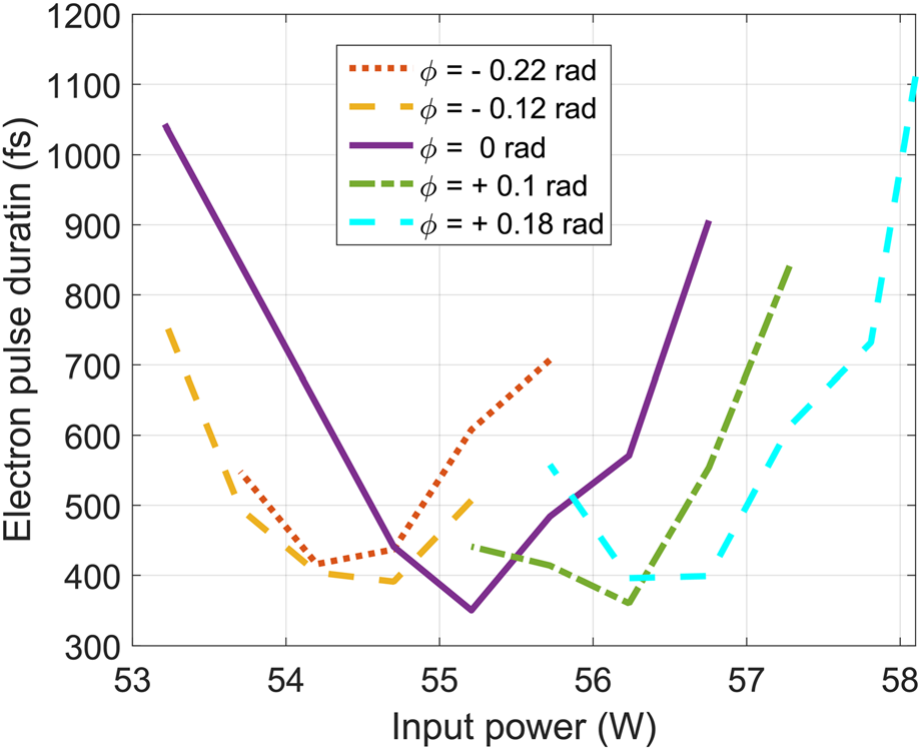
Electron pulse duration as a function of the RF cavity input power at different phases of the cavity electric field for pulses containing $5\times 10^{5}$ electrons.

Figure 5 shows the pulse duration as a function of the RF cavity input power for different phases of its electric field, for pulses containing $5\times 10^{5}$ electrons. Phase zero corresponds to the phase where the electric field crosses zero when the electron pulse is at the center of the cavity. The optimal compression is achieved at the phase zero, where the applied force is symmetric in time and the average kinetic energy of the electron pulse does not change. The pulse duration was measured in an accumulation mode where the signal is averaged over 5 s, corresponding to 25 000 electron pulses. The resulting measurement is a convolution of the electron pulse duration and the time of arrival jitter between the laser and electron pulses. We have reached a resolution of 350±8 fs FWHM by optimizing the amplitude and phase of the cavity, which is consistent with previous results using similar systems.^48–50^ The pulse duration and uncertainty were retrieved from a set of 30 measurements with 500 ms integration time each.

The main factor limiting the temporal resolution of the current setup is the time of arrival jitter between the laser and electrons at the sample, which is caused by phase jitter in the RF signal. Based on numerical simulations using the General Particle Tracer code, we expect that the electron pulse duration on the target should be 250 fs for 5 × 10^5^ electrons and 100 fs for 10^5^ electrons. However, we have observed that the temporal resolution does not improve from 350 fs when reducing the charge from 5 × 10^5^ to 10^5^ electrons, which suggests a large contribution from the timing jitter. The timing jitter could be reduced by adding an additional feedback loop after the microwave amplifier that drives the RF compression cavity. If successful, the temporal resolution would approach the electron pulse duration, which can be less than 100 fs.

## IV. DIFFRACION MEASUREMENTS

We demonstrate the capability of the setup to capture diffraction patterns with a short integration time using two molecules, Trifluoroiodomethane (CF_3_I) and Nitrogen (N_2_). In order to get accurate interatomic distances from the diffraction patterns, the conversion from detector pixels to the scattering angle must be calibrated. We used the data from CF_3_I to calibrate the detector and then applied this calibration to extract the bond length from the diffraction pattern of N_2_.

The intensity of the diffraction pattern on the detector, for a gas of randomly oriented molecules, can be written as^51^

$$I_{Total}\left(s\right)=I_{0}\sum_{i=1}^{N}\sum_{j=1}^{N}f_{i}\left(s\right)f_{j}^{*}\left(s\right)\frac{\;\text{sin}\left(r_{ij}s\right)}{r_{ij}s},$$ (1)

where $I_{0}$ is a constant, s=2k sin (θ/2) is the magnitude of the momentum transfer, θ is the scattering angle, k=2π/λ is the wavenumber of the electrons, λ is the de Broglie wavelength, $r_{ij}$ is the distance between atoms i and j, $f_{i}\left(s\right)$ is the scattering form factor of the *i*th atom, and the double summations are over all atoms in the molecule.

The total scattering intensity can be split into an atomic and a molecular contribution

$$I_{Total}\left(s\right)=I_{Atomic}\left(s\right)+I_{Molecular}\left(s\right),$$ (2a)

$$I_{Atomic}\left(s\right)=I_{0}\sum_{i=1}^{N}{\left|f_{i}\left(s\right)\right|}^{2},$$ (2b)

IMolecular(s)=I0∑i=1 N∑j=1j≠iN|fi(s)||fj(s)| cos[ηi(s)−ηj(s)] sin(rijs)rijs, (2c)

where $\left|f_{i}\left(s\right)\right|$ and $\eta_{i}$ are the amplitude and phase of the form factor of the *i*th atom, respectively. $I_{Molecular}$ contains the structural information in the form of interference between the scattering of different atoms in the molecule, while $I_{Atomic}$ depends only on the atomic scattering amplitudes and does not contain structural information. The interference pattern can be better visualized using the modified molecular scattering sM defined by

$$sM=\frac{\left(I_{Total}-I_{Atomic}\right)s}{I_{Atomic}}=\frac{s\;I_{Molecular}}{I_{Atomic}}.$$ (3)

The radial distribution function $f_{r}(r)$, which has a peak corresponding to the interatomic distance $r_{ij}$ between each atom pair, is calculated by taking a sine-transform of sM

$$f_{r}\left(r\right)=\int_{0}^{s_{Max}}sM\left(s\right)\;\text{sin}\;\left(rs\right)e^{-\alpha s^{2}}ds,$$ (4)

where $s_{Max}$ is the maximum measured value of *s*, and *α* is a damping constant that is adjusted to minimize edge effects in the transform.

**FIG. 6.**
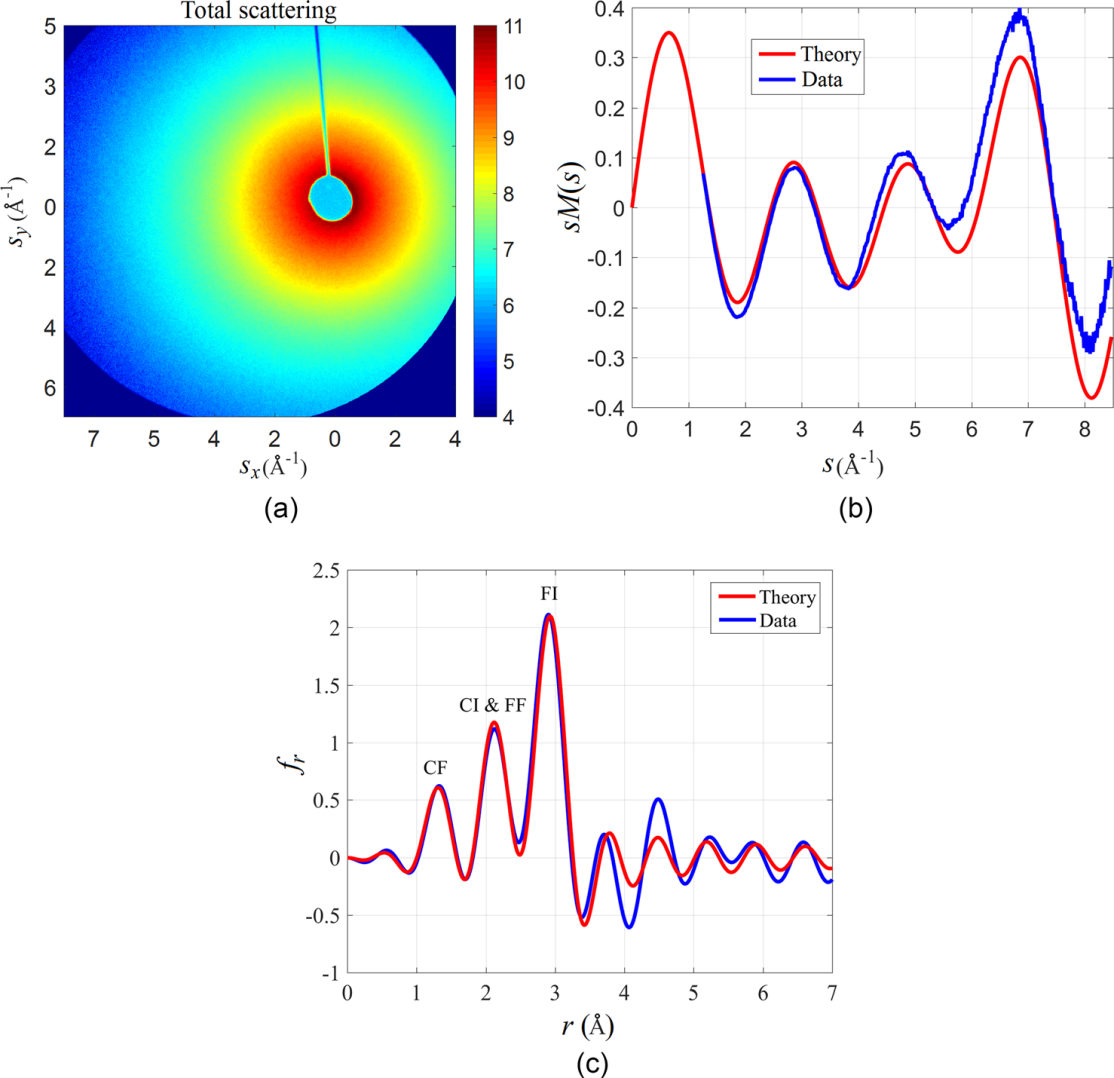
(a) Detected diffraction pattern of CF_3_I by the low noise CCD camera on a logarithmic scale. The data are gathered over 20 s exposure time. (b) Azimuthal average of sM in Equation (3) from the detected intensity in (a). (c) Real space reconstruction of the interatomic distances.

Figure 6(a) shows the measured diffraction pattern of CF_3_I on a logarithmic scale. The image shows that there is a significant number of scattered electrons even at the largest diffraction angles captured by the screen. The diffraction was acquired in a single frame with an integration time of 20 s, with a beam current of 70 pA ($∼10^{5}$ electrons/pulse). The diameter of the gas jet was 220 *μ*m and the backing pressure on the nozzle was 140 Torr. Based on the ratio of scattered to incident electrons and the known atomic scattering factors,^52^ we estimate the gas density in the interaction region to be $1.6\times 10^{16}$ molecules/cm^3^. An image with the gas jet turned off was also acquired and used for background subtraction. The electron beam was purposely placed off-center to increase the *s*-range of the measurement. A beam stop was used to block the directly transmitted electron beam.

The sM and $f_{r}$ are extracted from the diffraction patterns using standard data analysis methods for gas electron diffraction.^53^ The total intensity is azimuthally averaged and filtered to remove high frequency noise. The background is removed by fitting an exponential function to the zeros of the theoretical sM function. To calibrate the detector, the fitting process is repeated for different values of the pixel number to s conversion factor of the detector, until the minimum difference between the experiment and the theory is found. Figure 6(b) shows the azimuthally averaged sM(s) obtained from the diffraction pattern in Figure 6(a). There is good agreement between the theory and the experiment after the calibration. Figure 6(c) shows the corresponding $f_{r}$, which also shows a close match between the theory and the experiment. The peaks correspond to the different interatomic distances in the molecule, $r_{\text{FI}}=2.89\;Å$, $r_{\text{FF}}=2.15\;Å$, $r_{\text{CI}}=2.14\;Å$, and $r_{\text{CF}}=1.33\;Å$. These results compare favorably with previous UGED experiments with CF_3_I,^28^ where the integration time required for a single diffraction pattern was on the order of 1 h for comparable gas densities, due to the lower electron beam current.

**FIG. 7.**
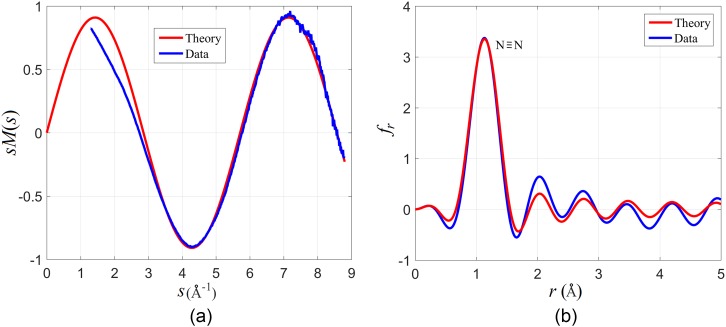
(a) Experimental and theoretical sM for Nitrogen. (b) The real space reconstruction retrieved from the sM in part (a).

We have used the calibration obtained with CF_3_I to extract the N-N bond length. The diffraction pattern was captured with a total integration time of 20 s with a beam current of 70 pA. The backing pressure behind the nozzle was 700 Torr, and the gas jet had a diameter of approximately 200 *μ*m in the interaction region. The density in the interaction region was estimated to be $1.6\times 10^{17}$ molecules/cm^3^. Figure 7(a) shows the experimental and theoretical sM for N_2_, and Figure 7(b) shows the corresponding $f_{r}$. In both cases, there is good agreement between the experiment and the theory. We have retrieved a bond length of 1.093 ± 0.013 Å, which agrees with the known value of 1.098 Å. The bond length was retrieved by simulating the sM for different values of the bond length and finding the value that best fits the experimental data. The uncertainty represents the standard deviation in a set of measurements taken under the same conditions. Figure 7(b) shows that the position of the peak in $f_{r}$ overlaps very well in the experiment and theory. The FWHM of the peak is 0.4 Å, which becomes important for retrieving the structure of molecules where there are multiple closely spaced distances.

It is important to note that one of the bottlenecks in UGED was that integration times on the order of an hour were needed to achieve a sufficient SNR for structure retrieval. Previous UGED experiments with keV electrons reached a resolution of 850 fs, with an average beam current of 3 pA,^28^ while recent UGED experiments with MeV electrons have reached a resolution of 200 fs with an average beam current of 0.7 pA. Our setup approaches the temporal resolution achieved with MeV electrons, but with a two order of magnitude increase in the beam current. For pump-probe experiments where many diffraction patterns are needed, this will be a significant advantage in how many pump-probe delays can be probed. In addition, the high beam current will be essential for investigating more complex molecules with low vapor pressure, where the target density can be one or two orders of magnitude lower.

## V. CONCLUSION

A new table-top setup was designed and built for UGED experiments that can reach femtosecond resolution with a high average beam current. The setup uses an RF cavity to compress the electron pulses and a tilted laser pulse to compensate for the group velocity mismatch. We have shown that the device can compress electron pulses with up to half a million electrons; however, we have found the best configuration for UGED experiments to be pulses with $∼10^{5}$ electrons/pulse (average beam current of 70 pA at the repetition rate of 5 kHz) which can be compressed to a duration of 350 fs and delivered on the target with a small divergence angle and a transverse diameter of 300 *μ*m FWHM to match the size of the gas jet. There is a tradeoff between the number of electrons, pulse duration, and the beam divergence, where any parameter can be improved at the expense of the others. The RF compression broadens the energy spread of the electron pulses, so there is an additional tradeoff between the minimum compressed electron pulse duration and the energy spread. Based on numerical simulations, we expect that the energy spread for 10^5^ electrons will be below 1%. An energy spread of a few percent does not significantly affect the quality of the diffraction patterns, but for pulses with more than a million electrons the energy spread may become an issue. Currently, the *s*-range that can be captured by this setup is limited by the size of the phosphor screen to 6.9 Å^−1^ with the beam at the center of the screen, which we increased to 8.8 Å^−1^ by moving the beam off-center, as shown in Figure 6(a). We are planning to add a magnetic lens after the sample to refocus the diffraction pattern, such that the *s*-range can be adjusted for each experiment. This flexibility will allow us to fully optimize the system depending on the temporal and spatial scales relevant to a given molecular reaction.

## References

[1] H. Mark and R. Wierl , Naturwissenschaften 18, 205 (1930). 10.1007/BF01494849

[2] L. O. Brockway and L. Pauling , Proc. Natl. Acad. Sci. U.S.A. 19, 860 (1933). 10.1073/pnas.19.9.860 16577584 PMC1086202

[3] P. Goodman , *Fifty Years of Electron Diffraction: In Recognition of Fifty Years of Achievement by the Crystallographers and Gas Diffractionists in the Field of Electron Diffraction* ( D. Reidel Publication Co., 1981).

[4] R. Henderson , Q. Rev. Biophys. 28, 171 (1995). 10.1017/S003358350000305X 7568675

[5] T. L. Leggett and D. A. Kohl , J. Chem. Phys. 59(2), 611–616 (1973). 10.1063/1.1680065

[6] A. A. Ischenko *et al.*, Appl. Phys. B: Lasers Opt. 32, 161 (1983). 10.1007/BF00688823

[7] D. L. Monts *et al.*, Appl. Spectrosc. 41, 631 (1987). 10.1366/0003702874448580

[8] W. L. Faust , J. D. Ewbank , D. L. Monts , and L. Schafer , Rev. Sci. Instrum. 59, 550 (1988). 10.1063/1.1139885

[9] J. D. Ewbank *et al.*, Rev. Sci. Instrum. 63, 3352 (1992). 10.1063/1.1142552

[10] V. A. Lobastov , J. D. Ewbank , L. Schäfer , and A. A. Ischenko , Rev. Sci. Instrum. 69, 2633 (1998). 10.1063/1.1148991

[11] J. C. Williamson and A. H. Zewail , Proc. Natl. Acad. Sci. U.S.A. 88, 5021 (1991). 10.1073/pnas.88.11.5021 11607189 PMC51799

[12] J. C. Williamson , M. Dantus , S. B. Kim , and A. H. Zewail , Chem. Phys. Lett. 196, 529–534 (1992). 10.1016/0009-2614(92)85988-M

[13] J. D. Geiser and P. M. Weber , in *SPIE's 1995 International Symposium on Optical Science, Engineering, and Instrumentation, International Society for Optics and Photonics* (1995), pp. 136–144.

[14] M. Ben-Nun , T. J. Martínez , P. M. Weber , and K. R. Wilson , Chem. Phys. Lett. 262, 405 (1996). 10.1016/0009-2614(96)01108-6

[15] H. Ihee , J. Cao , and A. H. Zewail , Chem. Phys. Lett. 281, 10 (1997). 10.1016/S0009-2614(97)01167-6

[16] J. C. Williamson , J. Cao , H. Ihee , H. Frey , and A. H. Zewail , Nature 386, 159 (1997). 10.1038/386159a0

[17] J. Cao , H. Ihee , and A. H. Zewail , Chem. Phys. Lett. 290, 1 (1998). 10.1016/S0009-2614(98)00520-X

[18] J. Cao , H. Ihee , and A. H. Zewail , Proc. Natl. Acad. Sci. U.S.A. 96, 338 (1999). 10.1073/pnas.96.2.338 9892634 PMC15137

[19] H. Ihee *et al.*, Science 291, 458 (2001). 10.1126/science.291.5503.458 11161194

[20] R. Srinivasan , V. A. Lobastov , C. Y. Ruan , and A. H. Zewail , Helv. Chim. Acta 86, 1761 (2003). 10.1002/hlca.200390147

[21] R. Srinivasan , J. S. Feenstra , S. T. Park , S. Xu , and A. H. Zewail , Science 307, 558 (2005). 10.1126/science.1107291 15637234

[22] J. S. Baskin and A. H. Zewail , ChemPhysChem 6, 2261 (2005). 10.1002/cphc.200500331 16273580

[23] A. H. Zewail , Annu. Rev. Phys. Chem. 57, 65 (2006). 10.1146/annurev.physchem.57.032905.104748 16599805

[24] B. J. Siwick , J. R. Dwyer , R. E. Jordan , and R. J. D. Miller , Science 302, 1382 (2003). 10.1126/science.1090052 14631036

[25] R. Ernstorfer , M. Harb , C. T. Hebeisen , G. Sciaini , T. Dartigalongue , and R. J. D. Miller , Science 323, 1033 (2009). 10.1126/science.1162697 19164708

[26] G. Sciaini *et al.*, Nature 458, 56 (2009). 10.1038/nature07788 19262668

[27] G. Sciaini and R. J. D. Miller , Rep. Prog. Phys. 74, 096101 (2011). 10.1088/0034-4885/74/9/096101

[28] C. J. Hensley , J. Yang , and M. Centurion , Phys. Rev. Lett. 109, 133202 (2012). 10.1103/PhysRevLett.109.133202 23030087

[29] J. Yang , J. Beck , C. J. Uiterwaal , and M. Centurion , Nat. Commun. 6, 8172 (2015). 10.1038/ncomms9172 26337631

[30] M. S. Robinson , P. D. Lane , and D. A. Wann , Rev. Sci. Instrum. 86, 013109 (2015). 10.1063/1.4905335 25638074

[31] J. C. Williamson and A. H. Zewail , Chem. Phys. Lett. 209, 10 (1993). 10.1016/0009-2614(93)87193-7

[32] S. P. Weathersby *et al.*, Rev. Sci. Instrum. 86, 073702 (2015). 10.1063/1.4926994 26233391

[33] J. Yang *et al.*, Faraday Discuss. 194, 563 (2016). 10.1039/C6FD00071A 27711826

[34] J. Yang *et al.*, Nat. Commun. 7, 11232 (2016). 10.1038/ncomms11232 27046298 PMC4822053

[35] J. Yang *et al.*, Phys. Rev. Lett. 117, 153002 (2016). 10.1103/PhysRevLett.117.153002 27768362

[36] T. Van Oudheusden *et al.*, J. Appl. Phys. 102, 093501 (2007). 10.1063/1.2801027

[37] T. Van Oudheusden *et al.*, Phys. Rev. Lett. 105, 264801 (2010). 10.1103/PhysRevLett.105.264801 21231672

[38] M. Gao *et al.*, Nature 496, 343 (2013). 10.1038/nature12044 23598343

[39] V. R. Morrison *et al.*, Science 346, 445 (2014). 10.1126/science.1253779 25342797

[40] R. P. Chatelain , V. R. Morrison , B. L. Klarenaar , and B. J. Siwick , Phys. Rev. Lett. 113, 235502 (2014). 10.1103/PhysRevLett.113.235502 25526134

[41] R. J. D. Miller , Annu. Rev. Phys. Chem. 65, 583 (2014). 10.1146/annurev-physchem-040412-110117 24423377

[42] P. Zhang , J. Yang , and M. Centurion , New J. Phys. 16, 083008 (2014). 10.1088/1367-2630/16/8/083008

[43] P. Baum and A. H. Zewail , Proc. Natl. Acad. Sci. U.S.A. 103, 16105 (2006). 10.1073/pnas.0607451103 17056711 PMC1637544

[44] G. H. Kassier *et al.*, Rev. Sci. Instrum. 81, 105103 (2010). 10.1063/1.3489118 21034115

[45] O. Zandi , K. J. Wilkin , and M. Centurion , “ Implementation and modeling of a femtosecond laser-activated streak camera,” Rev. Sci. Instrum. (submitted). 10.1063/1.4985008 28667974

[46] J. Yang , O. Zandi , P. Zhang , and M. Centurion , Proc. SPIE 9198, 91980N (2014). 10.1117/12.2061100

[47] O. J. Luiten , S. B. Van der Geer , M. J. De Loos , F. B. Kiewiet , and M. J. Van Der Wiel , Phys. Rev. Lett. 93, 094802 (2004). 10.1103/PhysRevLett.93.094802 15447108

[48] R. P. Chatelain , V. R. Morrison , C. Godbout , and B. J. Siwick , Appl. Phys. Lett. 101, 081901 (2012). 10.1063/1.4747155

[49] M. Gao *et al.*, Opt. Express 20, 12048 (2012). 10.1364/OE.20.012048 22714191

[50] M. Gao , Y. Jiang , G. H. Kassier , and R. J. D. Miller , Appl. Phys. Lett. 103, 033503 (2013). 10.1063/1.4813313

[51] I. Hargittai and M. Hargittai , *Stereochemical Applications of Gas-Phase Electron Diffraction* ( Wiley VCH, 1988).

[52] A. Jablonski , F. Salvat , and C. J. Powell , *NIST Electron Elastic-Scattering Cross-Section Database, Version 3.1, Standard Reference Data Program Database 64, US Department of Commerce* ( National Institute of Standards and Technology, Gaithersburg, MD, 2003).

[53] H. Ihee , B. M. Goodson , R. Srinivasan , V. A. Lobastov , and A. H. Zewail , J. Phys. Chem. A 106, 4087 (2002). 10.1021/jp014144r

